# Prevention and management of CMV infection in pediatric solid organ transplant recipients

**DOI:** 10.3389/fped.2023.1098434

**Published:** 2023-02-20

**Authors:** Shanthi S. Balani, Sanober Sadiq, Chelsey J. Jensen, Sarah J. Kizilbash

**Affiliations:** ^1^ Division of Nephrology, Department of Pediatrics, University of Minnesota, Minneapolis, MN, United States; ^2^ Division of Nephrology, Department of Pediatrics, University of California, San Francisco, CA, United States; ^3^ Department of Solid Organ Transplant, University of Minnesota, Minneapolis, MN, United States

**Keywords:** opportunistic infections, pediatric solid organ transplant, cytomegalovirus treatment, cytomegalovirus prevention, cytomegalovirus emerging treatment

## Abstract

Human cytomegalovirus (CMV) remains one of the most common opportunistic infections following solid organ transplantation in children. CMV causes morbidity and mortality through direct tissue-invasive disease and indirect immunomodulatory effects. In recent years, several new agents have emerged for the prevention and treatment of CMV disease in solid organ transplant recipients. However, pediatric data remain scarce, and many of the treatments are extrapolated from the adult literature. Controversies exist about the type and duration of prophylactic therapies and the optimal dosing of antiviral agents. This review provides an up-to-date overview of treatment modalities used to prevent and treat CMV disease in solid organ transplant (SOT) recipients.

## 1. Introduction

**Table 1:** Guidelines/recommendations per the American society of transplantation of infectious disease in regards to prevention are (3).

1. Antiviral prophylaxis may be given to any at-risk patients to prevent CMV disease after solid organ transplantation. 2. It should be started within ten days after transplant 3. Valganciclovir is the preferred agent 4. Duration based on serostatus: • If Donor+/Recipient−: Antiviral prophylaxis for 6 months for kidney transplant recipients • If Recipient+: Antiviral prophylaxis for 3 months for kidney transplant recipients • If Donor−/Recipient−: Antiviral prophylaxis not recommended 5. Use of CMV-specific T-cell immune measures to guide the duration of antiviral prophylaxis has been suggested but is still under investigation 6. Preemptive therapy may be used for effective prevention of CMV disease in solid organ transplant recipients. 7. Preemptive therapy is effective for prevention of CMV disease in D+/R− liver and kidney transplant recipient as long as close surveillance and follow up is available 8. The duration of preemptive therapy should be individualized with CMV QNAT or pp65 antigenemia measured weekly 9. There is widely applicable viral threshold to guide initiation of antiviral therapy but should be assay-specific, center-specific and risk specific 10. Antiviral therapy should be continued until viremia is no longer detectable and is below a predefined threshold.

**Table 2:** Antiviral medications for CMV prevention and treatment in pediatric SOT.

Medication	Prophylaxis	Treatment	Considerations
Ganciclovir (I.V.)	5 mg/kg I.V. once daily	5 mg/kg I.V. every 12 h	Dose adjustments needed for CrCl <70 ml/min/1.73 m^2^
Valganciclovir (oral)	Dose (mg): 7 x BSA^a^ x CrCl^b^ once daily Alternative^c^: 14–17 mg/kg once daily	Dose (mg): 7 x BSA x CrCl twice daily	Max single dose: 900 mg Dose adjustments needed for CrCl < 60 ml/min/1.73 m^2^
Valacyclovir (oral)	Adult: 2,000 mg 4 times per day Pediatric: dosing has not been established	Not recommended	Dose adjustments needed for CrCl < 50 ml/min/1.73 m^2^
Maribavir (oral)	Not recommended	Patients ≥12 years AND ≥35 kg: 400 mg twice daily^d^	May increase concentrations of common immunosuppression (calcineurin and mTOR inhibitors)
Foscarnet (I.V.)	Not recommended	60 mg/kg I.V. every 8 h OR 90 mg/kg I.V every 12 h	Second- or third-line option for resistant CMV Nephrotoxic Dose adjustments needed for kidney impairment
Cidofovir (I.V.)	Not recommended	5 mg/kg once weekly x 2, then every 2 weeks thereafter in combination with Probenecid	Third-line option for resistant CMV Nephrotoxic Dose adjustments needed for kidney impairment

^a^ Body surface area calculated using Mosteller body surface area equation.

^b^ Creatinine clearance calculation based on modified Schwarz formula. Max CrcL of 150 ml/min/1.73 m^2.^

^c^ Off-label dosing.

^d^ For treatment of refractory CMV.

Cytomegalovirus (CMV) is one of the most frequent infections seen in solid organ transplant recipients. The pediatric population is specifically at an increased due to the high prevalence of seronegativity in children and seropositivity in donors (1). CMV infection in post-transplant period can be acquired as a primary infection including transmission from allograft in case of a serologically positive donor, a re-infection with a different strain of CMV or reactivation of the virus from latency following a primary infection. With the advent of potent antiviral agents, the incidence of invasive CMV disease has decreased; however, it continues to cause morbidity and mortality through its indirect immunomodulatory effects, resulting in increased rates of rejection, chronic allograft injury, secondary opportunistic infections, and malignancies (2, 3). Furthermore, it is associated with bronchiolitis obliterans and chronic lung allograft dysfunction in lung recipients and coronary vasculopathy in heart recipients (4–6). This review provides an overview of the various modalities used to prevent and treat CMV disease in pediatric solid organ transplant (SOT) recipients.

### 2. Prevention strategies

#### 2.1. Prophylactic vs. preemptive therapy

Antiviral prophylaxis and pre-emptive therapy are two strategies employed to prevent invasive CMV disease in SOT recipients (Table 1). Prophylaxis therapy involves antiviral administration to all at-risk patients from the time of transplant to at least 3–6 months post-transplant or longer. In contrast, preemptive therapy includes close monitoring for CMV viremia and prompt initiation of antiviral therapy if CMV is detected (7).

Valganciclovir and oral/IV ganciclovir are commonly used for CMV prophylaxis. Valacyclovir is an alternate agent used in kidney transplant recipients (8). A randomized clinical trial including 372 D+/R− kidney, heart, and lung transplant recipients showed comparable CMV disease rates between ganciclovir and oral valganciclovir treated transplant recipients (9). For intermediate risk patients (Donor+/Recipient+or Donor−/Recipient+) prophylaxis is recommended for 3 months in kidney, heart and liver transplant recipients and 6–12 months in lung transplant recipients. Prophylaxis is typically not recommended in case of low risk patients (Donor−/Recipient−) (3). Prophylactic therapy is also recommended during treatment of acute rejection in solid organ recipients, especially if using lymphocyte-depleting therapy (3).

The optimal duration of CMV prophylaxis is unclear. A randomized clinical trial including Donor+/Recipient− kidney transplant recipients documented the incidence of CMV disease to be 36.8% in patients with 100 days of antiviral prophylaxis and 16.1% in those with 200 days of prophylaxis therapy, indicating the importance of longer duration of prophylaxis in those at higher risk of CMV disease (10). The risk of CMV infection/disease is the highest among lung, intestine, and vascularized composite tissue allograft recipients (3). In these patients, it may be reasonable to use a longer duration of antiviral prophylaxis (>12 months) (11). Furthermore, CMV immunoglobulins may be used for additional protection, particularly in lung transplant recipients; however, data on the efficacy of immunoglobulins for the prevention of CMV disease are limited (12, 13).

The preemptive therapy includes close surveillance of CMV viremia and prompt initiation of treatment with valganciclovir or oral ganciclovir when viremia is detected. Monitoring can be performed with either pp65 antigenemia or DNAemia (14). CMV quantitative nucleic acid testing (QNAT) calibrated with the World Health Organization International Reference Standard and reported as IU/ml is the preferred method for guiding preemptive therapy and monitoring treatment response. Variability still exists amongst different CMV QNAT assays, therefore, there is no widely applicable viral threshold for which to initiate preemptive therapy. This threshold should be defined by each transplant center and be assay- and risk-specific3. The duration of therapy is guided by viral load monitoring and is continued until CMV becomes undetectable or falls below the predefined viral load threshold (15). Valganciclovir has greater bioavailability than oral ganciclovir, making it the drug of choice for pre-emptive therapy (16). The major benefit of preemptive therapy is the prevention of delayed onset CMV-disease, greater antibody neutralization and CD 8 T-cell response (17, 18).

The safety of preemptive therapy has not been established for all solid organ transplants. Given the paucity of data and the highest risk of CMV disease, preemptive therapy is not recommended for lung, intestine, and vascularized composite tissue allograft recipients. The guidelines also list preemptive therapy as the less preferred therapy in heart recipients (3).

Both antiviral prophylaxis and preemptive therapy are associated with risks and benefits. While antiviral prophylaxis prevents early CMV viremia and is reported to improve long-term graft survival (19, 20), it is associated with myelotoxicity, increased cost, and greater incidence of late-onset CMV disease (21, 22). In contrast, preemptive therapy prevents drug toxicity and minimizes costs by targeting only those who are at the highest risk of invasive CMV disease. Since it requires close monitoring and poses logistical challenges, concerns exist about the use of preemptive therapy in high risk patients. CMV drug resistance has been observed with both strategies (23). Some centers have adopted a hybrid approach using antiviral prophylaxis for 3–6 months, followed by close surveillance of CMV viremia.

#### 2.2. Monoclonal antibodies

There is evidence that neutralizing antibodies have a role in preventing CMV disease. Compared to polyclonal immunoglobulins, monoclonal antibodies are more target specific, have greater potency, and less toxicity (24). A phase 2 randomized, double-blind, placebo-controlled trial, including 138 Donor+/Recipient− kidney transplant recipients receiving a combination of two monoclonal antibodies (RG7667), demonstrated a reduced incidence of CMV disease, delayed time to CMV viremia and no significant side effects with the use of monoclonal antibodies (25).

#### 2.3. Immune surveillance

Identification of Donor+/Recipient− CMV transplant recipients who are at highest risk for having CMV disease using a laboratory marker will offer a more selective and appropriate use of prophylaxis, leading to better transplant outcomes. Studies have shown that patients with adequate T-cell immunity have a lower risk of CMV viremia and disease (26). CMV-specific peptide-based enzyme linked immunosorbent spot (ELISPOT) detects the release of interferon gamma from effector cells, primarily CD4 and CD8 T cells. A prospective international multicenter observational study in 368 kidney transplant recipients (260 Donor+/Recipient− and 277 Recipient+) from 43 centers showed that CMV events were significantly lower in assay positive vs. assay negative patients (cutoff value of >40 sfu/2.5 × 105 cells) (27). The challenges for its clinical use are lack of routine availability, high cost, and slow turnaround time.

### 3. Treatment

The treatment of CMV in pediatric SOT recipients (Table 2), whether for asymptomatic viremia or CMV disease, is largely derived from the adult literature. Cautious reduction in immune suppression and treatment with antiviral agents remain the mainstay for treatment of CMV infection in children. Although oral ganciclovir may be appropriate as an initial choice in asymptomatic viremia or mild/moderate disease, it is no longer widely available in the United States. IV ganciclovir is the preferred agent in more severe CMV disease or if there is a concern for impaired GI absorption (3, 28). Other antiviral agents such as foscarnet and cidofovir are considered second or third-line options, mainly reserved for use in cases of ganciclovir resistance due to their highly nephrotoxic potential. Data is emerging regarding newer antiviral agents, letermovir and maribavir, in both prevention and treatment of refractory CMV infection in the adult and pediatric SOT literature. Duration of treatment for CMV disease is dependent on resolution of clinical symptoms treatment guidelines. Following completion of treatment, many centers will provide secondary prophylaxis for 1–3 months to reduce the risk for recurrence, although not currently supported by high quality evidence (3).

### 3.1. Ganciclovir/valganciclovir

Ganciclovir was the first antiviral approved for the treatment of CMV infection in 1989. Ganciclovir and its oral pro-drug valganciclovir, compete with deoxyguanosine triphosphate for binding to viral DNA polymerase, thereby inhibiting viral DNA synthesis (29). Several studies have demonstrated efficacy of ganciclovir and valganciclovir for the treatment of CMV in adult SOT recipients, and this is largely extrapolated to the pediatric SOT population (30–32). Hematologic toxicities such as leukopenia, neutropenia, thrombocytopenia, and anemia are well-known and reversible adverse effects of ganciclovir and valganciclovir (33). These agents are recommended to be used with caution in patients with pre-existing cytopenias, and use should be avoided when ANC < 500 cells/ml, platelet count is <25,000/ml, or hemoglobin <8 g/dl. However, disseminated CMV can itself suppress the bone marrow, and granulocyte-colony stimulating factor (G-CSF) may be used to manage bone marrow suppression in patients requiring prolonged treatment with ganciclovir or valganciclovir.

FDA-approved dosing for both ganciclovir and valganciclovir in the pediatric population has undergone scrutiny in recent years. In 2009, valganciclovir received pediatric approval for the prevention of CMV in kidney and heart transplant recipients using an FDA approved dosing algorithm of 7 x BSA x CrCl (calculated from the modified Schwartz formula) (29). In 2010, the FDA issued a safety alert cautioning valganciclovir overexposure in pediatric patients with low body weight, low body surface area, and below normal serum creatinine levels as the labeled dosing algorithm did not account for these patient factors (29). With this alert, valganciclovir labeling was revised to include a maximum creatinine clearance of 150 ml/min/1.73 m^2^ and a maximum dose not to exceed 900 mg. Overall this has led to alternative weight-based and BSA-based dosing strategies and an overall lack of consensus amongst providers (34). A national survey published in 2018 found that only 25% of transplant centers in the United States that participated in the survey utilized the FDA-approved dosing algorithm, many of them using modified variations of the formula or different dosing strategies alltogether (34). Several small, single-center pediatric studies have gone on to support the findings that the FDA-approved dosing algorithm leads to supratherapeutic levels (AUC12/24 > 60 mcg*h/ml) in the majority of pediatric patients (35, 36), whereas weight based dosing demonstrated subtherapeutic exposure (36, 37), however, more recent large pediatric data suggests that this may not impact clinical outcomes of prevention of CMV viremia (38). Another small retrospective study found no difference in the development of CMV viremia by weight-based vs. FDA-labeled dosing; however, the incidence of neutropenia was higher in those who received FDA-labeled dosing (39). Finally, there are several, recent population pharmacokinetic model-based studies which have looked at IV ganciclovir dosing algorithms in pediatric patients and found that standard dosing of ganciclovir 5 mg/kg daily (prevention dose) or every 12 h (treatment dose) are projected to achieve subtherapeutic ganciclovir concentrations, particularly in younger patients <2 years old. These findings led to the development of a new dosing algorithm (3x BSA x CrCL), which has been reviewed by the European Medicine Agency (EMA) and added to ganciclovir prescribing information in Europe (37).

Ganciclovir and valganciclovir resistance can occur in up to 2%–4% of pediatric patients with CMV infection (28). Pediatric SOT recipients are a unique population due to high likelihood of CMV mismatch (CMV D+/R−) at the time of transplantation (28). The most common gene mutation in patients initially treated with ganciclovir or valganciclovir is the UL97 kinase mutation which occurs in 90% of cases, with the UL54 DNA polymerase gene mutation typically evolving later. Drug resistance should be suspected in patients who develop refractory CMV infection after prolonged antiviral drug exposure and in those failing to respond after at least 2 weeks of appropriate therapy (3, 28).

### 3.2. Cidofovir/foscarnet/brincidofovir

Treatment of ganciclovir-resistant CMV disease continues to remain a challenge. Apart from cautious reduction in immunosuppression, foscarnet is the first choice for treatment of ganciclovir-resistant CMV infection (3, 40, 41). Both foscarnet and cidofovir are effective against CMV isolates with isolated UL97 mutations, however there may be cross-resistance in the case of UL54 mutations especially with cidofovir. Genotypic assays should be used to guide therapy. Both are highly nephrotoxic, which remain the main concern with their use. Brincidofovir, a synthetic oral analogue of cidofovir with good oral bioavailability and reduced nephrotoxicity, although promising in preclinical studies, had disappointing results in phase 3 clincal study (42).

### 3.3. Maribavir

Maribavir is a CMV enzyme pUL97 kinase inhibitor that was FDA-approved in November 2021 for the treatment of post-transplant refractory CMV infection in both adult and pediatric patients ≥12 years of age weighing ≥35 kg (43). Maribavir is dosed 400 mg orally twice daily with no dose adjustments for kidney impairment, and lacks bone marrow and kidney toxicity (29). It may be swallowed whole, crushed, or dispersed and administered through a feeding tube if needed, providing administration options often needed in pediatric patients. The most common side effect reported includes dysgeusia. It can potentially increase drug concentrations of substrates of Cytochrome P450 3A4 and/or P-glycoprotein (P-gp), including tacrolimus, cyclosporine, everolimus, and sirolimus. In addition, Maribavir may antagonize the antiviral activity of ganciclovir and valganciclovir, therefore, co-administration is not recommended.

Maribavir's FDA approval was largely based on the SOLSTICE trial (44), a randomized, open-label, multicenter trial with maribavir 400 mg twice daily or investigator-assigned therapy (IAT; valganciclovir/ganciclovir, foscarnet, or cidofovir) for 8 weeks in 352 HSCT and SOT recipients with refractory CMV infections. Significantly more patients in the maribavir group achieved CMV viremia clearance at 8 weeks than patients in the IAT group (55.7% vs. 23.9%). A greater proportion of patients with genotypic resistance to IAT achieved viremia clearance at 8 weeks vs. IAT group (62.8% vs. 20.3%). Secondary outcomes including CMV viremia clearance and symptom control was maintained through week 16. Recurrence, however, was common after discontinuation (50% in maribavir and 39% in IAT group) as noted in additional label information (29). Interestingly, non-published label data reported from the SOLSTICE study also showed development of treatment-emergent resistance with pUL97 substitutions that were detected in 58 patients (47 patients who were on-treatment failures and 11 patients who were relapsed). Maribavir had less acute kidney injury compared to foscarnet (8.5% vs. 21.3%) and less neutropenia compared to valganciclovir/ganciclovir (9.4% vs. 33.9%). SOLSTICE trial design intended to include patients age ≥12 years, however, did not in fact enroll any patients under 18. Labelling approval for use in patients 12 years and older (and over 35 kg) largely is derived from population pharmacokinetic modeling.

Maribavir has also been examined for use in preemptive treatment (45). In a dose finding, randomized open-label trial in adult patients with HSCT or SOT, maribavir appeared to have similar efficacy to valganciclovir for clearing CMV viremia at 3 and 6 weeks. It is important to note that 2 patients in this study developed CMV recurrence after initial response to treatment and were found to have developed resistance mutation in the UL97 protein kinase.

Maribavir appears to be an appealing option allowing for potential outpatient management of patients with refractory or resistance CMV while sparring adverse effects like myelotoxicity. More data, however, is needed to evaluate the clinical impact and potential for cross-resistance with increased use of Maribavir.

### 3.4. Letermovir

Letermovir is novel CMV DNA terminase enzyme complex inhibitor (pUL51, pUL56, pUL89) FDA approved for the prevention of CMV infection and disease in adult CMV-seropositive recipients of allogeneic HSCT (29). Letermovir has emerged as an attractive therapeutic option given its unique mechanism of action for potential use in multi-drug resistant CMV and due to its lack of hematologic and kidney toxicities. Letermovir is not currently approved for use in SOT or in the pediatric population, and most recent guidelines do not support routine use at this time (3, 28). Letermovir, however, has been used off-label for both treatment and secondary prophylaxis of CMV infections in SOT, mostly in cases of multi-drug resistant CMV. Case reports and case series in the SOT population have shown mixed results (46–50) and emergence of resistance and treatment failure have been reported (46, 51). In a large multicenter series, letermovir was examined for patterns of off-label use in the management of CMV infection in 47 patients and included patients down to 15 years of age (50). The most common indications for letermovir use were intolerance to other agents, resistance, and preference for use of an oral agent. In most patients treated with letermovir, low levels of viremia (<1,000 IU/ml) were achieved prior to transition to letermovir. Viral suppression was maintained; however, the authors comment that it is unknown whether the treatment success was due to letermovir or other factors such as immune suppression reduction or spontaneous viral clearance. Success rates were lower for patients with higher viral loads (>1,000 IU/ml). There is currently an ongoing, phase II trial examining the safety and efficacy of letermovir and will include children (age ≥12 years) (NCT03728426).

Doses utilized in case reports and case series have ranged from 240 mg daily (when co-administered with cyclosporine) to 960 mg daily (47–51). FDA recommended dose for prevention of CMV in HCST is 480 mg daily, highlighting the need for more formal treatment dose studies. Most common adverse effect reported in clinical trials is gastrointestinal toxicity (diarrhea, nausea, vomiting), others include peripheral edema, fatigue and headache. Letermovir is a moderate inhibitor of cytochrome P450 3A4 and an empiric tacrolimus dose reduction of 30% in addition to therapeutic drug monitoring has been suggested (52). Cyclosporine may also increase the concentration of letermovir; therefore, initial letermovir dosing should be 240 mg daily when used concomitantly.

### 3.5. Adjuvant therapies

Cytomegalovirus immune globulin (CMVIG) or IVIG has been used as an adjunctive therapy and may be considered for use in patients with life-threatening disease or those with treatment resistant CMV (3, 28). Much of the evidence in SOT is limited to small uncontrolled trials and case series conducted prior to modern antiviral era (53–55). Newer evidence has found that the use of CMVIG as rescue therapy (in addition to antiviral therapy and immune suppression reduction) appears to be effective at controlling viral replication in the thoracic transplantation (56–59). Based on the lack of controlled studies, treatment with CMVIG in the adult and pediatric patient population remains limited to resistant infection or complicated cases (59). There is no consensus on the optimal dose and frequency, and pediatric doses range from 100 to 400 mg/kg with varying published frequencies (29). Infusion reactions and high cost or accessibility can often be limiting factors for use.

### 3.6. Adoptive T-cell therapy (ATC)

Adoptive transfer of CMV viral specific T-cells (VST) is an emerging therapeutic option for treatment of refractory and resistant CMV (3, 28). It aims to restore virus-specific T cell immunity facilitating reduction in CMV viral loads and improvement or resolution of CMV disease. CMV specific T-cells can be donor-derived (allogenic) or isolated from the transplant recipient (autologous). VST therapy has been primarily studied in HSCT population and found to be safe and effective (60–63). Studies in SOT population are limited, especially among the pediatric population. A prospective phase I clinical trial included 13 SOT recipients (4 kidney, 8 lung, 1 heart) with ganciclovir-resistant CMV infection showed that 11 of the 13 patients (84%) responded with complete resolution or reduction in viremia, end-organ disease, and/or cessation or reduction of antiviral drug use (64). Several other case reports using allogenic VST therapy in kidney (65) and liver (16 year old) (66) and autologous therapy in lung (67) transplant recipients have shown promising results. VST therapy in the pediatric HSCT population has been successfully reported in a recipient with drug resistant CMV retinitis (68). There are several ongoing studies in adult and pediatric patients to evaluate VST therapy in the management of CMV in SOT recipients (NCT04331275, NCT03950414, NCT02532452, NCT02779439). It is important to note that isolation of VST is time and labor-intensive, limited to a few specialized facilities or treatment centers. There has been increased interest in development of “off the shelf” third party banked cells to help overcome this process (28).

## 4. Vaccines

Development of CMV vaccine to prevent CMV infection, especially among high-risk SOT recipients (D+/R−) has been underway for several decades; however, there is no currently available vaccine for clinical use. Several vaccines have been evaluated in phase I and phase II clinical trials in solid organ transplant recipients with mixed results and there are numerous vaccine candidates in development (69).

## 5. Conclusion/future directions

While the discovery of newer antiviral agents and the prospects of a vaccine offer hope, pediatric data on the safety and efficacy of several therapies remains scarce, requiring extrapolation from adult studies. Additional research is needed to determine appropriate dosing, the optimal duration of prophylactic therapy, and the risk-benefit analysis of the newer therapies for the pediatric SOT population.

## Author contributions

All authors contributed to the article and approved the submitted version.

## Conflict of interest

The authors declare that the research was conducted in the absence of any commercial or financial relationships that could be construed as a potential conflict of interest.
